# DEGRO practical guideline for central nervous system radiation necrosis part 2: treatment

**DOI:** 10.1007/s00066-022-01973-8

**Published:** 2022-08-29

**Authors:** Denise Bernhardt, Laila König, Anca-L. Grosu, Stefan Rieken, Sandro M. Krieg, Wolfgang Wick, Benedikt Wiestler, Friederike Schmidt-Graf, Felix Sahm, Jens Gempt, Bernhard Meyer, Bernd J. Krause, Cordula Petersen, Rainer Fietkau, Michael Thomas, Frank Giordano, Andrea Wittig-Sauerwein, Jürgen Debus, Ghazaleh Tabatabai, Peter Hau, Joachim Steinbach, Stephanie E. Combs, Expert Panel of the German Society of Radiation Oncology (DEGRO)

**Affiliations:** 1 Klinik und Poliklinik für Radioonkologie und Strahlentherapie, Klinikum rechts der Isar, Technische Universität München, Ismaninger Straße 22, 81675 Munich, Germany; 2 Institute of Radiation Medicine (IRM), Department of Radiation Sciences (DRS), Helmholtz Zentrum München (HMGU), Ingolstädter Landstraße Ingolstädter Landstraße 1, 85764 Oberschleißheim, Germany; 3 Partner Sites Munich, Deutsches Konsortium für Translationale Krebsforschung (DKTK), Freiburg and Heidelberg, Germany; 4 Klinik für Radioonkologie und Strahlentherapie, Universitätsklinikum Heidelberg, Im Neuenheimer Feld 400, 69120 Heidelberg, Germany; 5 Heidelberger Ionenstrahltherapie-Zentrum (HIT), Im Neuenheimer Feld 450, 69120 Heidelberg, Germany; 6 Heidelberg Institute of Radiation Oncology (HIRO), Im Neuenheimer Feld 400, 69120 Heidelberg, Germany; 7 Clinical Cooperation Unit Radiation Oncology, German Cancer Research Center (DKFZ), Im Neuenheimer Feld 280, 69120 Heidelberg, Germany; 8 National Center for Tumor diseases (NCT), 69120 Heidelberg, Germany; 9 Department of Radiation Oncology, Medical Center—University of Freiburg, Faculty of Medicine, University of Freiburg, Robert-Koch-Str. 3, 79106 Freiburg, Germany; 10 Department of Neuroradiology, Technical University Munich, School of Medicine, Klinikum rechts der Isar, 81675 Munich, Germany; 11 Clinic of Radiotherapy and Radiation Oncology, University Medical Center Göttingen, Robert-Koch-Str. 40, 37075 Göttingen, Germany; 12 Clinical Cooperation Unit Neurooncology, German Consortium for Translational Cancer Research (DKTK), German Cancer Research Center (DKFZ), 69120 Heidelberg, Germany; 13 Department of Neurology and Neurooncology Program, National Center for Tumor Diseases, Heidelberg University Hospital, 69120 Heidelberg, Germany; 14 Department of Neurosurgery, Technical University Munich, School of Medicine, Klinikum rechts der Isar, Munich, Germany; 15 Department of Neurology, Technical University Munich, School of Medicine, Klinikum rechts der Isar, Munich, Germany; 16 Department of Neuropathology, University Hospital Heidelberg and CCU Neuropathology, German Consortium for Translational Cancer Research (DKTK), German Cancer Research Center (DKFZ), 69120 Heidelberg, Germany; 17 Department of Nuclear Medicine, Rostock University Medical Centre, 18057 Rostock, Germany; 18 Department of Radiotherapy and Radiation Oncology, University Medical Center Hamburg-Eppendorf, Martinistraße 52, 20246 Hamburg, Germany; 19 Department of Radiation Oncology, University Hospital Erlangen, Friedrich-Alexander-Universität Erlangen-Nürnberg, 2306, Erlangen, Germany; 20 Comprehensive Cancer Center Erlangen-European Metropolitan Region of Nuremberg (CCC ER-EMN), 91054 Erlangen, Germany; 21 Department of Thoracic Oncology, Thoraxklinik at Heidelberg University Hospital, 69120 Heidelberg, Germany; 22 Department of Radiation Oncology, University Hospital Bonn, 53127 Bonn, Germany; 23 Department of Radiotherapy and Radiation Oncology, University Hospital Jena, Bachstraße 18, 07743 Jena, Germany; 24 Department of Neurosurgery, University Hospital Tuebingen, Eberhard Karls University Tuebingen, 72076 Tuebingen, Germany; 25 Center for Neuro-Oncology, Comprehensive Cancer Center Tuebingen Stuttgart, University Hospital Tuebingen, Eberhard Karls University of Tuebingen, 72076 Tuebingen, Germany; 26 Department of Neurology, Eberhard Karls University of Tuebingen, 72076 Tuebingen, Germany; 27 Department Interdisciplinary Neuro-Oncology, Eberhard Karls University of Tuebingen, 72076 Tuebingen, Germany; 28 Wilhelm Sander-NeuroOncology Unit and Department of Neurology, University Hospital Regensburg, 93053 Regensburg, Germany; 29 Dr Senckenberg Institute of Neurooncology, University Hospital, 60325 Frankfurt am Main, Germany; 30 Member of the German Center for Lung Research (DZL), Translational Lung Research Center Heidelberg (TLRC-H), 69120 Heidelberg, Germany; 31 National Center for Tumor Diseases (NCT), Heidelberg, Germany 69120

**Keywords:** Radiation necrosis, Stereotactic radiotherapy, Bevacizumab, Steroids, Brain metastases, Glioma, Blood–brain barrier disruptions

## Abstract

**Purpose:**

The Working Group for Neurooncology of the German Society for Radiation Oncology (DEGRO; AG NRO) in cooperation with members of the Neurooncological Working Group of the German Cancer Society (DKG-NOA) aimed to define a practical guideline for the diagnosis and treatment of radiation-induced necrosis (RN) of the central nervous system (CNS).

**Methods:**

Panel members of the DEGRO working group invited experts, participated in a series of conferences, supplemented their clinical experience, performed a literature review, and formulated recommendations for medical treatment of RN, including bevacizumab, in clinical routine.

**Conclusion:**

Diagnosis and treatment of RN requires multidisciplinary structures of care and defined processes. Diagnosis has to be made on an interdisciplinary level with the joint knowledge of a neuroradiologist, radiation oncologist, neurosurgeon, neuropathologist, and neurooncologist. If the diagnosis of blood–brain barrier disruptions (BBD) or RN is likely, treatment should be initiated depending on the symptoms, location, and dynamic of the lesion. Multiple treatment options are available (such as observation, surgery, steroids, and bevacizumab) and the optimal approach should be discussed in an interdisciplinary setting. In this practice guideline, we offer detailed treatment strategies for various scenarios.

## Introduction

Brain metastases (BM) are frequent in cancer patients and diagnosed in approximately 20 to 30% of all cancer patients during the course of their disease [1–3]. In the past decade, several innovations in cancer therapy have led to an improvement in outcomes. By prolonged survival, the absolute risk of developing BM as well as RN rises. Pseudoprogression and RN are frequent in glioma patients following radiotherapy and often the differentiation between true RN and pseudoprogression is challenging. RN is considered as a dose-limiting toxicity for radiotherapy (RT), especially in areas with critical structure involvement, such as the brainstem [4, 5]. Currently, there is no defined guideline for the treatment and diagnosis of RN. Several guidelines already recommend the use of steroids and bevacizumab in the treatment of RN, although there are no defined treatment algorithms [6, 7]. This lack of consensus was identified by the DEGRO and, therefore, the DEGRO board mandated the DEGRO Working Group for Neurooncology (AG NRO) to establish a practice guideline. In 2020, a position paper about the use of bevacizumab and the treatment of RN was already established and published by the DEGRO society. A detailed nomenclature of treatment-related changes and a multistep approach for their diagnosis was presented in part I of the DEGRO practice guideline. In this practice guideline, we have integrated the limited results from contemporary clinical trials and the available retrospective data. The guideline aims to provide guidance for treatment decisions. The implementation of this guideline requires multidisciplinary structures of care and defined processes of diagnosis and treatment of RN.

## Methods

This guideline was prepared by an expert panel of the DEGRO AG NRO in cooperation with members of the Neurooncological Working Group of the DKG-NOA. The guidelines’ subcommittee recruited a panel of recognized experts from the field of neurosurgery, neuroradiology, neuropathology, and neurooncology/neurology. This task force represents all disciplines involved in the diagnosis and care of patients with CNS RN/BBD. We retrieved references published in English on PubMed with the search terms “radiation necrosis” alone and in combination with “avastin,” “bevacizumab,” “steroids,” “radiosurgery,” “stereotactic,” “re-irradiation,” “vascular endothelial growth factor (VEGF),” “immunotherapy,” and “dexamethasone” from January 1, 2000, to November 1, 2021. We also identified publications through searches of the authors’ own files. Screening and initial eligibility were addressed by six authors (DB, SC, AG, SR, SK, and LK), consulting others for disagreement resolution. Panel members of DEGRO and experts participated in a series of virtual conferences, circular emails, supplemented their clinical experience, and formulated recommendations for the treatment and diagnosis of RN in clinical routine. The treatment recommendations were formed by full consensus of the participating experts.

### Treatment options

Several therapeutic approaches have been suggested in the past, including drug therapy, surgery, hyperbaric oxygen, heparin, warfarin, pentoxifylline, and vitamin E [8].

#### Corticosteroids

Corticosteroids, such as dexamethasone, have long been the gold standard of RN treatment. For symptomatic patients, corticosteroids are the first-line treatment due to their anti-inflammatory potential and reduction of BBB leakage [9]. Improvement can often be seen rapidly due to the reduction of edema in RN patients with symptoms associated with the edema. Corticosteroids are potent, but offer only symptomatic relief from RN. Corticosteroids can be a double-edged sword, with a significant benefit and a low incidence of adverse effects for a limited treatment duration, but unmindful withdrawal or prolonged administration can have serious side effects [10]. Adverse effects of corticosteroids are both dose and time dependent, whereas other adverse effects follow a linear dose response, with an incidence increase with higher dose. Other adverse effects may follow a threshold dose–response pattern [11]. Benefits of corticosteroid use include their general and fast availability in most countries and the relative inexpensiveness compared to bevacizumab use or surgery. Since there are no studies regarding dose, we prefer to use dexamethasone with an initially high starting dose of 20–40 mg intravenous dexamethasone or 8 mg (1-0‑0 up to 1‑1-1) in symptomatic patients for 3 to 5 days, followed by a gradual reduction. We recommend a short-course maintenance dose of 1.5–2 mg, until the first follow-up (FU) MRI (6–8 weeks after initial diagnosis of blood–brain barrier disruptions [BBD]/RN) is performed to prevent a rebound effect. For immediate symptom relief, an attempt with high-dose dexamethasone (e.g., 20 mg i.v.) can be reasonable. In general, the lowest possible dose for the shortest possible duration is recommended. Patient education is vital in recognizing the adverse effects early. Furthermore, communication with other health professionals, especially in post-hospital treatment, is necessary to ensure that the patient is adequately monitored and the corticosteroid dose is reduced. With multimodal oncological treatments, it is often necessary to combine dexamethasone with other ongoing systemic therapies. In the era of immunotherapy, this becomes challenging, posing the question of whether the immunosuppressive effects of corticosteroids may lower the efficacy of immune- or targeted therapies [12].

#### Bevacizumab

Bevacizumab is a potent mediator in inhibiting the vicious cycle of RN development, and the efficacy of bevacizumab is supported by class IIb evidence. A double-blind, placebo-controlled phase II study evaluated bevacizumab for the treatment of symptomatic and progressive CNS radiation necrosis [13]. Eligible bevacizumab-naïve patients had undergone cranial radiation for grade 2–3 primary brain neoplasm, meningioma, or head and neck carcinoma and had progressive neurological symptoms with radiographic evidence of radiation necrosis. Patients were randomized to receive bevacizumab 7.5 mg/kg every 3 weeks (*n* = 5) or placebo (*n* = 7). At week 6, a median increase of 14% in T2-weighted FLAIR volume was observed in placebo-treated patients vs. a median decline of 59% for bevacizumab-treated patients (*p* = 0.0149). In addition, a median increase of 17% in T1-weighted contrast-enhanced volume was observed in placebo-treated patients vs. a median decrease of 63% in bevacizumab-treated patients (*p* = 0.0058). Xu et al. investigated, in a multicenter open-label study, patients with RN who were randomly assigned to a bevacizumab group (5 mg/kg intravenously every 2 weeks, 4 cycles) or a corticosteroid group: 38 patients in the bevacizumab group showed a response, which was significantly higher proportion than in the corticosteroid group (65.5% vs. 31.5%, *p* < 0.001). Furthermore, radiographic response was improved in the bevacizumab group [14]. Several additional studies reported excellent response rates for the treatment of RN with bevacizumab. The recurrence rate for RN after bevacizumab treatment is unclear and studies have reported recurrence rates between 10 and 39%, mainly independent of the primary disease [15]. Li et al. indicated that the duration from RN diagnosis to start of bevacizumab is a predictive factor for RN recurrence [16]. In general, RN diagnosis is difficult, even more so in the recurrent setting. Therefore, accurate recurrence rates are rare because physicians are often unable to distinguish between RN and tumor progression. In addition to diagnostic response, bevacizumab offers clinical improvement in the majority of patients suffering from RN with minimal toxicity [15]. The dose in the literature varies, but lower doses of 5.0–7.5 mg/kg every 2 weeks seem sufficient under regular control of blood pressure or thrombotic events to prevent typical side effects. The obvious broad therapeutic range shows that studies are needed to establish a minimum dose requirement for achieving the maximum clinical benefit and to make the bevacizumab treatment more cost effective. In a prospective clinical trial, patients were even treated with ultra-low doses of 1 mg/kg and radiographic responses were observed in 20 of the 21 patients [17], suggesting that efficacy is associated with its anti-angiogenic effects rather than dose [14, 18]. Several studies reported that side effects were mild and reported mostly low-grade adverse events [13, 17, 19]. Side effects included vascular events, hypertension, fatigue, proteinuria, anemia, leukopenia, neutropenia, and lymphocytopenia. In a prospective trial, Levin et al. reported that 6 (55%) patients experienced serious adverse events, including aspiration pneumonia, pulmonary embolism, and superior sagittal sinus thrombosis [13]. Another prospective trial by Xu et al. reported 40 grade 1 or 2 adverse events experienced by 58 patients but only one grade 3 adverse event of ischemic stroke. Interestingly, comparable side effects were seen in the corticosteroid-treated group, indicating that bevacizumab may not increase toxicity in the treatment of RN compared to dexamethasone [14]. The results of a randomized phase II study, which aims to investigate whether the addition of bevacizumab to standard corticosteroid therapy results in greater improvement in symptoms and less treatment-induced symptoms compared to standard corticosteroid therapy for patients with symptomatic RN following SRS are pending (NCT02490878) [20].

#### Laser interstitial thermal therapy

Laser interstitial thermal therapy (LITT) is a potential treatment option, but direct comparative data are scarce, with only a few patients being treated with LITT, and the technique is not generally available [21, 22]. The advantage of LITT is its minimal invasiveness and ability to provide same-procedure pathologic confirmation of RN. Palmisciano summarized the available evidence in a recent review. Among the 18 studies included, 143 patients received bevacizumab and 148 underwent LITT. Both strategies were effective in providing post-treatment symptomatic improvement (*p* = 0.187), weaning off steroids (*p* = 0.614), and local lesion control (*p* = 0.5) [23].

#### Hyperbaric oxygen therapy

Hyperbaric oxygen therapy (HBOT) demonstrated the ability to reduce edema and prospective studies report radiographic and symptomatic improvement of RN with HBOT [24–27]. Approximately 90% of the patients improved and 60% responded clinically during the course of HBOT after a median of 30 treatments. It has to be highlighted that in many reported cases, HBOT was administered with concomitant dexamethasone or bevacizumab, and studies evaluating hyperbaric oxygen therapy alone are rare and the available evidence is low. Common side effects of HBOT are usually low grade and can include ear barotrauma, dyspnea, ear pain requiring myringotomy, and sinusitis [24, 27, 28].

#### Surgical removal of rapidly progressing cerebral radiation necrosis in brain metastases

Standard of care for rapidly progressing, symptomatic RN is considered to be surgical removal, depending on the location and estimated morbidity of the procedure. This approach is consistent with the tumor-like growth pattern of RN and can be achieved with low rates of morbidity if patients are selected carefully and discussed within an interdisciplinary setting [29]. The incidence of wound healing and surgical complications, including serious and fatal complications, is increased in bevacizumab-treated patients; thus, at least 28 days of bevacizumab “wash-out” before elective surgery is recommended at present [30]. Currently, data on the benefit of surgical resection of RN are sparse. However, existing evidence shows that gross total resection leads to reduced edema and use of steroids during follow-up [31]. Preoperative symptoms, such as headache or seizures, only improved in 55% after surgery but were independent of the extent of resection. However, regarding BM data, symptom relief was reported by 55% of patients undergoing RN resection independently of the extent of surgical resection (EOR), while most effects of bevacizumab were only reported 3 months after treatment or later.

#### Surgical removal of rapidly progressing cerebral radiation necrosis in glioma

GBM does not usually have extracranial disease necessitating systemic therapy but requires an evaluation of rather a purely local disease.

Surgery can potentially reduce the CNS tumor burden if RN is combined with residual tumor cells. Yet, at least for GBM, no difference was shown in overall survival for surgery or bevacizumab if RN consisted of necrosis only vs. residual tumor cells [32]. Thus, there seems to be no advantage of cytoreduction or RN resection in terms of survival. Considering the goal of maximizing quality of life (QOL) in tumor patients, the treatment of agonizing symptoms, such as seizures or headache, is a legitimate rationale for surgical treatment. The resection of RN is very similar to a glioma resection, with a continuous border between healthy tissue and necrosis. Thus, the same principles need to be applied by the neurosurgeon, including intraoperative neurophysiology via MRI to secure maximum safe resection. Since RN neighbors previous resection cavities, it can affect eloquent subcortical anatomy, necessitating the respective caution and measures, especially considering the usually closer location to eloquent tracts.

### Considerations for the treatment of radiation necrosis in the era of immunotherapy

Corticosteroids are one of the principal treatments for immune-related adverse events of checkpoint inhibitors (CPIs) but also an exclusion criterion for CPI clinical trials [33–35]. In day-to-day clinical routine, corticosteroids are often used as part of the palliative treatment for cancer-related symptoms such as dyspnea and edema in symptomatic brain metastases [36, 37]. In a recent review, Petrelli et al. summarized the data of studies where the outcome of corticosteroid-using patients treated with immunotherapy were compared with those not assuming or using steroids at lower doses (less than 10 mg equivalent of prednisone) [38]. They found that patients taking steroids were at an increased risk of limited overall survival and progression compared to patients not using steroids (*p* = 0.03). Even though immune-related toxicities necessitate corticosteroid therapy for improvement, use of steroids in these cases seems not to reduce overall survival (OS) in cancer patients treated with CPIs and may be safely administered without compromising outcome [33, 39]. Conversely, more caution is needed for patients using higher doses of corticosteroids for a longer period of time, especially as palliative treatment. In analogy to this, short-course steroid use for immediate symptom relief in patients with symptomatic RN (e.g., 20 mg dexamethasone i.v.) might not compromise CPI treatment, but long-term steroid use over a course of several weeks might be detrimental. In these cases, discussing different treatment options (e.g., bevacizumab or surgery) may avoid compromising treatment with CPIs. Dexamethasone, as the potentially cheaper treatment of RN compared to bevacizumab or surgery, might compromise not only the survival of patients undergoing a working CPI treatment but also might be rendered less cost effective by inhibiting the effect of cost-intensive CPIs. CPIs have significantly improved the prognosis of patients with various tumors. As part of their multidisciplinary treatment, many patients will be treated with high-dose radiotherapy (RT) to CNS metastases and receive CPIs either concurrently or within short time intervals both before or after RT; this combination has been observed to beneficially decrease the incidence of new CNS metastases [3]. On the contrary, CPIs and other targeted therapies have been demonstrated to enhance the risk for symptomatic RN [4]. Most RN occur within the first year after RT [40]. Despite the general notion of preferably no or low steroid doses while receiving CPI, most reported RN has been treated with steroids, while few authors have reported the efficacy of bevacizumab [41, 42]. A current clinical phase I study is investigating the effect of low-dose radiosurgery in combination with CPI on the occurrence and course of RN [43].

### Reimbursement situation and consecutive workflow issues

Despite clear data supporting bevacizumab’s efficacy in the treatment of RN, it is still considered an off-label use. As a result, seeking insurance approvals can lead to treatment delays and, if ultimately not covered, the drug cost can be expensive for patients and treating physicians [44]. In order to avoid reimbursement claims following off-label use, physicians in Germany (and other European countries) can file an application for cost coverage with the patients’ health insurance prior to treatment initiation [45]. In this context, the position paper of the European Society for Medical Oncology (ESMO) describes a number of ethical problems for treating physicians [46]. Where evidence is available, several clinical practice guidelines recommend off-label use of drugs but this is perceived by the ESMO as “outside existing regulatory boundaries” [46] and by prescribing off-label, the physician is asked to take a special responsibility. Although this off-label use of drugs is not unpermitted or illegal, there are some important differences in comparison with licensed pharmaceutical products within the approved indications [47–49]. The off-label issue can be as acute as the symptoms of a patient with RN. For bevacizumab it is currently unlikely that pharmaceutical companies will support large-scale phase II/III trials. When the drug patent expires, there is limited financial interest in pursuing any label extension. In cases of acute RN, physicians have to decide between immediate start of a potentially curative therapy and risking reimbursement claims. Filing an off-label application often takes several weeks before reimbursement is granted. In patients with severe neurological symptoms and no surgical treatment options, any treatment delay can cause irreversible neurological brain damage or even be lethal. Additionally, in centers with high volumes of stereotactic RT or re-irradiation cases, reimbursement applications become a time-consuming and staff-intensive task which is not financially compensated for.

## Proposed treatment strategy

Treatment of BBD and RN is similar, although it should be performed more straightforwardly in RN due to the more aggressive and tumor-like clinical behavior. Treatment should be initiated based on symptomatology and the anatomical region of the CEL. Observation (on a regular basis every 3 months) is a viable option in asymptomatic patients with classical BBD that occur as a side effect after RT, since they are mostly self-limiting and temporary. Also, patients who have been treated with SRS sometimes experience an increase in size during FU. In patients receiving immunotherapy, delayed CEL is possible, an effect that is already well known and considered in the iRECIST and RANO criteria [5, 50]. However, it is recommended to more closely monitor patients clinically and radiologically (e.g., every 2–3 months). Of further importance is the fact that once there is a progressive RN detected, this damage can be irreversible, underlining the importance of prompt treatment induction with bevacizumab after diagnosis. Since bevacizumab is effective in reduction of edema, corticosteroids should be reduced as soon as possible. A FU-MRI scan should be scheduled after four cycles to evaluate further treatment with bevacizumab. Bevacizumab dose is typically 5–10 mg/kg, q2–4w, and patients receive at least two doses. Due to the half-time of bevacizumab of 3 weeks, treatment with only one application is not recommended, since it can cause rebound effects. Because the treatment goal is symptom oriented and not prolongation of survival, we suggest that patients should be treated until symptoms are relieved and imaging improves; the treatment should then be discontinued. The decision on whether to use bevacizumab or perform surgery should be discussed within tumor boards and put into the context of the underlying primary disease, especially in patients with extracranial tumor progression requiring immediate systemic therapy. The possible morbidity of each strategy and necessary perioperative systemic treatment interruptions should be weighed against the potential benefit. On the other hand, if bevacizumab is used, the further need of surgical interventions can be complicated. Detailed treatment strategies for glioma patients and patients with brain metastases are explained in Table 1 and 2, respectively.

**Table 1:** Proposed treatment algorithm for BBD and RN in glioma patients

Most likely radiographic and clinical diagnosis according to interdisciplinary decision	Symptomatic	Dynamic/progressive	Localization	Treatment strategy
SCE	No	Fluctuating or no	Every location	Observation with regular FU
BBD	No	No	Uncritical	Observation (regular FU MRI, approximately 12 weeks)
BBD	No	No	Critical	MRI follow-up (8–12 weeks), short-course dexamethasone can be considered depending on the size and extent of edema to prevent symptoms in critical areas like brainstem
BBD	No	Yes	Uncritical	In smaller lesions, regular observation (FU MRI approximately 12 weeks) is reasonable. For larger lesions short-course dexamethasone can be considered if severe edema is present to prevent symptoms followed by shorter MRI follow-up (6–8 weeks). Diagnostic verification of RN with FET PET MRI in progressing glioma lesions can be discussed
BBD	Yes, but not severe	No or yes	Uncritical	Short-course dexamethasone if severe edema is present can be considered, MRI follow-up (6–8 weeks)
BBD	Severe	No or yes	Uncritical or critical	For immediate symptom relief an attempt with dexamethasone (e.g., 20 mg i.v.) can be reasonable, followed by short-course dexamethasone for a few days depending on the symptoms; if no symptom improvement occurs, surgical intervention or bevacizumab should be considered (→ *continue treatment as RN, diagnostic verification of RN with FET PET MRI in progressing glioma lesions*)
RN	No	No	Uncritical or critical	MRI follow-up (8–12 weeks), short-course dexamethasone can be considered depending on the size and extent of edema and the location of the lesion (e.g., critical brainstem lesions) followed by shorter MRI or for glioma FET PET MRI follow-up (6–8 weeks)
RN	No	Yes	Uncritical	Short-course dexamethasone if edema is present, MRI or FET PET MRI follow-up (6–8 weeks)
RN	Yes, but not severe	Yes	Uncritical	Consider diagnostic verification of RN with FET PET MRI in progressing lesions in glioma. Consider short-course dexamethasone depending on the symptoms; if no symptom improvement is seen, surgical intervention or bevacizumab should be considered
RN	Yes, but not severe	Yes	Critical	Consider diagnostic verification of RN with FET PET MRI in progressing lesions. Consider a short course of dexamethasone for a few days depending on the symptoms and extent of the edema; if no symptom improvement is seen, surgical intervention or bevacizumab should be considered. In patients with no or only small edema, surgery or bevacizumab without prior dexamethasone should be considered
RN	Severe	Yes (rapidly progressing)	Uncritical or critical	Consider diagnostic verification of RN with FET PET MRI in progressing lesions. Surgical intervention or bevacizumab should be considered. In patients with no or only small edema, surgery or bevacizumab without prior dexamethasone therapy should be considered. For immediate symptom relief, an attempt with high-dose dexamethasone (e.g., 20 mg i.v. can be reasonable)

*IT* immunotherapy, *FU* follow-up, *RN* radiation necrosis, *SCEs* speckled contrast-enhancing lesions, *BBD* blood–brain barrier disruptions

**Table 2:** Proposed treatment algorithm for BBD and RN in patients with brain metastases

Most likely radiographic and clinical diagnosis according to interdisciplinary decision	Symptomatic	Dynamic/progressive	Localization	Treatment strategy
BBD	No	No	Uncritical	Observation (regular FU MRI, approximately12 weeks)
BBD	No	No	Critical	MRI follow-up (8–12 weeks), short-course dexamethasone can be considered depending on the size and extent of edema to prevent symptoms in critical areas like brainstem. In patients undergoing IT, MRI follow-up without dexamethasone should be considered
BBD	No	Yes	Uncritical	In smaller lesions regular observation (FU MRI approximately12 weeks) is reasonable. For larger lesions, short-course dexamethasone can be considered if severe edema is present to prevent symptoms, followed by shorter MRI follow-up (6–8 weeks). In patients undergoing IT, MRI follow-up without dexamethasone should be considered
BBD	Yes, but not severe	No or yes	Uncritical	Short-course dexamethasone can be considered if edema is present with MRI follow-up (6–8 weeks). In patients undergoing IT, dexamethasone should be critically discussed. In rapidly progressing lesions, bevacizumab can be considered
BBD	Severe	No or yes	Uncritical or critical	For immediate symptom relief, an attempt with dexamethasone (e.g., 20 mg i.v.) can be reasonable, followed by short-course dexamethasone for a few days depending on the symptoms; if no symptom improvement occurs, surgical intervention or bevacizumab should be considered. In symptomatic patients with no or only small edema and in patients undergoing IT, surgery or bevacizumab without prior dexamethasone should be considered. (*→ continue treatment as RN*)
RN	No	No	Uncritical or critical	MRI follow-up (8–12 weeks), short-course dexamethasone can be considered depending on the size and extent of edema and the location of the lesion (e.g., critical brainstem lesions) followed by shorter MRI follow-up (6–8 weeks). In patients undergoing IT, only MRI follow-up is recommended
RN	No	Yes	Uncritical	Short-course dexamethasone if edema is present, MRI follow-up (6–8 weeks). In patients undergoing IT, only MRI follow-up should be discussed depending on the dynamic. In rapidly progressing lesions, bevacizumab can be considered
RN	Yes, but not severe	Yes	Uncritical	Consider short-course dexamethasone depending on the symptoms; if no symptom improvement is seen, surgical intervention or bevacizumab should be discussed. In patients undergoing IT, surgery or bevacizumab should be considered
RN	Yes, but not severe	Yes	Critical	Consider a short course of dexamethasone for a few days depending on the symptoms and extent of the edema; if no symptom improvement appears, surgical intervention or bevacizumab should be discussed. In symptomatic patients with no or only small edema and in patients undergoing IT, surgery or bevacizumab without prior dexamethasone should be considered
RN	Severe	Yes (rapidly progressing)	Uncritical or critical	For immediate symptom relief an attempt with high-dose dexamethasone (e.g., 20 mg i.v. can be reasonable). Surgical intervention or bevacizumab should be considered. In symptomatic patients with no or only small edema and in patients undergoing IT, surgery or bevacizumab without prior dexamethasone should be considered

*IT* immunotherapy, *FU* follow-up, *RN* radiation necrosis, *BBD* blood–brain barrier disruptions

## Conclusion

Due to the increasing use of SRS and Re-RT, high-dose treatment at the skull base, and other dose-escalating radiotherapy approaches, detection of new or progressing CEL is encountered more frequently. Since BBD may be self-limiting and reversible, treatment is not always mandatory and clinical symptoms as well as radiographic features should be taken into consideration. On the other hand, the rapid progression and tumor-like growth patterns of RN as well as the accompanying clinical symptoms often require prompt initiation of treatment with corticosteroids, bevacizumab, or surgery. High-dose radiotherapy provides a curative treatment option in many situations, and RN may be an accepted side effect in this context. This practice guideline offers a multistep approach to improve treatment of radiation-induced injuries, helping us to treat unwanted side effects effectively, and offering a balanced recommendation for anticipated side effects associated with curative high-dose treatments.
